# Voiding Dysfunction Associated with Pudendal Nerve Entrapment

**DOI:** 10.1007/s11884-012-0156-5

**Published:** 2012-09-28

**Authors:** Marc Possover, A. Forman

**Affiliations:** 1 Department for Gynecology/Oncology & Neuropelveology, Hirslanden Clinic, Zürich, Switzerland; 2 Department of Gynecology & Neuropelveology, University of Aarhus, Aarhus, Denmark

**Keywords:** Pudendal neuralgie, Alcock’s canal syndrome, Sacral radiculopathies, Pudendal pain, Vulvodynia

## Abstract

Pudendal nerve entrapment (Alcock canal syndrome) is an uncommon source of chronic pelvic pain, in which the pudendal nerve is entrapped or compressed. Pain is located in the perineal, genital and perianal areas and is worsened by sitting. By simple entrapment of the PN without neurogenic damages, pain is usually isolated. In neurogenic damages to the PN, genito-anal numbness, fecal and/or urinary incontinence can occurred. PNE can be caused by obstetric traumas, scarring due to genitoanal surgeries (prolaps procedures!), accidents and surgical mishaps. Diagnosis is based on anamnesis, clinical examination including vaginal or rectal palpation of the pelvic nerves with selective nerve blockade. Pudendal pain non systematic mean PNE since other neuropathies may induce pudendal pain. So sacral radiculopathies (sacral nerves roots S#2-4) are underestimated etiologies frequently responsible for pudendal pain with irradiation in sacral dermatomes, bladder hypersensitivity or in neurogenic lesions, bladder retention.

## Introduction

Pudendal pain, chronic proctalgia, coccygodynia, vulvodynia pudendal neuralgia are all pain situations reported as “chronic pelvic pain”. These problems occur in 7 to 24 % of the population and are associated with impaired quality of life and high health care costs. Ano-perineo-genital pain represent frequent complaints usually as a result of common and easily recognizable organic disorders such as anal fistula, thrombosed haemorrhoids, genitoanal cancer or other dermatologic pathologies, but can also occur under circumstances in which no organic cause can be found. These pain syndromes are then poorly understood, with little scientific information available to guide their diagnosis and treatment. Pathologies of the pudendal nerve (PN) can be responsible for such pain and are well known by physicians and patients as they are accessible not only by symptomatic treatments but also by etiologic surgical treatment.

The Alcock’s canal syndrome (entrapment of PN at the Alcock’s canal) is the best well-known etiology for pudendal neuralgia but not the only one. Pathologies of the distal branches or the endopelvic portion of the PN, or even of the sacral nerves roots #2-4/5 (SNR) that contain pudendal afferents (S2/3/4) can also induce such pain. Endopelvic lesions are less well studied and because their diagnosis is difficult and the surgical approach challenging and invasive, these etiologies are mostly managed by symptomatic treatments. In addition to pain, these lesions may cause urinary dysfunction, which may be further affected by the medical analgetic treatment.

## Anatomical and Neurophysiological Considerations

The pudendal nerve is a sensory and somatic nerve which originates from the ventral rami of the second, third, and fourth (and occasionally the fifth) sacral nerves roots. After branching from the sacral plexus, the PN leaves the pelvis through the less sciatic foramen and travels three main regions: the gluteal region, the pudendal canal, and the perineum. It accompanies the internal pudendal vessels upward and forward along the lateral wall of the ischiorectal fossa, being contained in a sheath of the obturator fascia termed the pudendal canal (= Alcock’s canal). The pudendal nerve gives off three distal branches, the inferior rectal nerve, the perineal nerve and the dorsal nerve of the penis in males, corresponding to the dorsal nerve of the clitoris in females.

The PN innervates the external genitalia of both sexes, as well as sphincters for the bladder and the rectum. As the bladder fills, the pudendal nerve becomes excited. Stimulation of the pudendal nerve results in contraction of the external urethral sphincter. Contraction of the external sphincter, coupled with that of the internal sphincter, maintains urethral pressure (resistance) higher than normal bladder pressure. The storage phase of the urinary bladder can be switched to the voiding phase either involuntarily (reflexively) or voluntarily. The pudendal nerve then causes relaxation of the levator ani so that the pelvic floor muscle relaxes. The pudendal nerve also signals the external sphincter to open. The sympathetic nerves send a message to the internal sphincter to relax and open, resulting in a lower urethral resistance. The PN is also known to have a potential modulative effect on bladder function. Somatic afferent fibers of the pudendal nerve are supposed to project on sympathetic thoracolumbar neurons to the bladder neck and modulate their function. This neuromodulative effect works exclusively at the spinal level and appears to be at least partly responsible for bladder neck competence and at least continence [1].

Therefore, if a problem occurs within the nervous system, the entire voiding cycle is affected.

## Pudendal Neuralgia – Symptomatology

The diagnosis of pudendal neuralgia is reserved for patients who have allodynia in the entire distribution of the pudendal nerve - vulvar, perineal and perianal area – without any irradiation in other lumbosacral dermatomes and with typically severe pain on sitting, relieved in contrary by standing, and absent when recumbent or when sitting on a toilet seat. Various other symptoms may occur in some cases, for example urinary hesitancy (difficulty starting the flow of urine), frequency (frequent need to pass urine), urgency (sudden sensation to pass urine), constipation/painful bowel movements, reduced awareness of defecation (the process of passing bowel motions), sexual dysfunction including loss of libido, recurrent numbness of the penis and/or scrotum after prolonged cycling, altered sensation of ejaculation and impotence in men.

Symptoms of prostatitis-like pain are conducted through pudenal afferents and occur in 11 % of American men, and approximately 95 % of the patients diagnosed with chronic prostatitis have no evidence of bacterial infection or inflammatory cells in the prostatic fluid [2].

Further possible symptoms include burning, loss of sensation or numbness, increased sensitivity, electric shock or stabbing pain, knife-like or aching pain, feeling of a lump or foreign body, twisting or pinching, abnormal temperature sensations, constipation, pain and straining with bowel movements, straining or burning when urinating, painful intercourse, and sexual dysfunction – including hyperarousal or decreased sensitivity.

Neurological examination is then extremely important for diagnosis of nerve damages. Extrinsic lesions (PN entrapment) do not include any loss of sensation or feeling of numbness in pudendal dermatomes but on the contrary imply hyperesthesia without troubles of micturition or continence. In neurogenic PN damages, loss of sensation or numbness selectively is usually combined with contralateral anal deviation due to homolateral perineal/perianal myoatrophia; urodynamic testing can show bladder overactivity [3] but may also be normal. Urethral incontinence occurs only in bilateral neurogenic PN damages.

For diagnosis, behind the anamnesis, the more commonly used tests are the PN motor latency test (PNMLT), electromyography (EMG), diagnostic nerve blocks, and magnetic resonance neurography (MRN). EMG studies of the pudendal nerve, often touted as a diagnostic tool, are unreliable since they can be abnormal after vaginal delivery or vaginal hysterectomy and do not define the neurologic level of the pathology. Transvaginal, or in men transrectal palpation of the pudendal nerve at the sacrospinal ligament is the key to the diagnosis: patients have exquisite tenderness when digital pressure is applied over the pudendal nerve and produce a typically Tinel’s sign (sensation of tingling or “pins and needles” in the distribution of the PN). A selective PN block by transvaginal or perineal approach – a technique of anesthesia well known in obstetrics - is then crucial for the diagnosis and treatment of pudendal neuralgia.

## Pudendal Neuralgias – Etiologies

There are numerous possible causes for pudendal neuropathy. Some of the possible causes are an inflammatory or autoimmune illness, frequently interpreted as infection. After iatrogenic nerve damages, which are frequent in obstetrics and gynecology pudendal neuralgia is common, with etiologies such as compression of the nerve through a postpartal haematoma, fibrosis of the ischiorectal fossa, stretching of the nerve during delivery or surgical damages during transvaginal sacrospinous colpopexy [4]. More recent interventions using mesh material for sacrospinal fixation [5], sacro-colpopexy or rectopexy may also expose patients to risk for pudendal nerves damages [6].

## Differencial Diagnosis

### Distal Lesions of the Pudendal Branches.

The pain is neuropathic, mostly reported as allodynia located in a specific area of one of the distal branches of the pudendal nerve. This generally involves the dorsal branch (rectal branch) or the middle branch (perineal branch) when the lesion is secondary to an episiotomy or proctological procedure. A trigger point is found by palpation of the area of pain while vaginal/rectal palpation of PN is painless. Pelvic dysfunctions, especially neurogenic incontinences never occur. When sub dermal infiltration with local anesthesia produces significant improvement, infiltration with botulinum toxine is a promising treatment option which may control pain efficiently for several months.

### Genitofemoral Neuropathy.

When the genitofemoral nerve is affected, pain may be felt in the inguinal area with irradiation in the internal aspect of the thigh (never below the knee) and in the genital area. Lesions of the genital branch of the genitofemoral nerve induce vulvodynia or pudendal pain located selectively in the anterior portion of the vulva (clitoris). Surgical access to the inguinal region (appendectomy, herniorraphia, introduction of lateral trocar for laparoscopy…) exposes patients to risk for injuries to the genitofemoral nerve. Neurologic symptoms are then restricted to sensory changes except in injuries to the genitofemoral nerve in males that can also induce troubles or loss of cremastic reflex. Pain is located not only in the groin area and the ventral genital area (as in lesion of the ventral branch of the PN) but also in the internal aspect of the tight (lesion of the femoral branch), never below the knee. Since the genital branch is only sensitive, neither bladder dysfunctions nor urinary incontinence occur. Inguinal nerve blockade with anesthetic agent is then the method of choice for diagnosis.

### Contracture/Spasm of Bulbospongiosus Muscles.

In females, dyspareunia is located in the distal third of the vagina, increased by vaginal penetration. Vaginal palpation of the bulbospongiosus muscles is painful while the muscles are generally under massive tension forming like a “tente” in upper part of the vagina. Relaxation of the muscles can be easier obtained by botulinum toxine infiltration.

### Sacral Radiculopathies.

Incidences of sacral radiculopathies are widely underestimated obviously because of lack of awareness that such lesions may exist, lack of diagnosis and acceptance, and lack of declaration and report of such lesions. The most probable reasons for neglecting pelvic nerve pathologies in medicine are the complexity of the pelvic nerve system, the difficulties of etiologic diagnosis and - probably the main reason - the limitations in access to the pelvic nerves for neurophysiologic explorations and neurosurgical treatments. Neurosurgical procedures techniques are well established in nerve lesions of the upper limb but pelvic retroperitoneal areas and surgeries to the pelvic nerves are still unusual for neurosurgeons [7]. In one of our own series of 136 consecutive patients suffering from pudendal pain, only 18 had presented a true PN entrapment while all other patients were suffering from a sacral radiculopathy involving the pudendal fibers contained in S#2-4/5.

The semiology may include pudendal pain but frequently involves other different “pelvic symptoms” such as pelvic pain, dys/apareunia, troubles/loss of vesical and/or rectal sensation with “non-pelvic symptoms”. These include low-back-pain (lumbosacral trunk, L5), pudendal pain (S2-4), vulvo-vaginodynia and coccygodynia (S3-4), sciatica (L5-S2), distal pain, and/or abnormal sensations in the legs or buttock.

In massive lesions of the SNR (plexopathy), loss of strength in hip extension, knee flexion, and dorsal plantar flexion of the foot can be detected. Because the vesical parasympathetic nerves (pelvic splanchnic nerves) are contained in the SNR #3-5, vesical symptoms are quasi constant. In extrinsic nerves irritation, bladder hypersensitivity is quasi systematic, but also bladder overactivity is even not unusual. In neurogenic damages of the nerves, behind loss of sensitivity or even numbness in corresponding dermatomes, ultrasonography shows usually postvoid residual urine while urodynamic testing confirm detrusor hypo- or even atonia as well as a detrusor hyposensitivity and an augmentation of bladder capacity.

In surgically induced sacral radiculopathies, correlation of clinical information with the surgical steps of the procedure permit a precise anatomical localization of the neural lesion, which is essential for adapting the therapeutic strategy. Perineal procedures induce pathologies of the pudendal nerve, abdominal/laparoscopic procedures of the sacral nerve roots while vaginal surgeries can induce both [6].

When nerve injuries have occurred, laparoscopic exploration not only offers an anatomic and functional exploration of the nerves, but may also result in effective neurosurgical treatment using techniques of nerve(s) decompression or reconstruction also by the laparoscopic approach. In non-postsurgical damages, systematic laparoscopic exploration has showed that not only pelvic cancers can induce nerve damage. In addition, frequent pathologies, such as deeply infiltrating endometriosis, uterine myomas or ovarian processes, or retroperitoneal vascular abnormalities (vascular entrapment) and retroperitoneal fibrosis may induce pelvic neuropathies, that can be treated by laparoscopic nerve decompression/neurolysis. Moreover, neurogenic lesions are accessible to laparoscopic implantation of electrodes for neuromodulation of the SNR [8]. Laparoscopy is therefore the essential and logical step in the management of pelvic nerve pathologies that must be indicated as soon as possible, before the nerve damage becomes irreversible and before the process of “pain chronification” starts.

### Piriformis Syndrome

The greater sciatic notch provides egress for the piriformis muscle. The pudendal nerve exits the pelvis at the inferior aspect of this muscle. In the athlete, flexion and abduction of the thigh are common motions, and they may lead to hypertrophy of the piriformis muscle. If the sciatic notch is narrowed because of the posterior orientation of the ischial spine, the cross-sectional area of the greater sciatic notch is reduced. Concomitant hypertrophy of the piriformis muscle may cause compression of the pudendal nerve against the posterior edge of the sacrospinous ligament. The piriformis syndrome is diagnosed primarily on the basis of symptoms and on the physical exam. There are no tests that accurately confirm the diagnosis, but MRI may be necessary to exclude other pathologies. Behind medical treatments and physiotherapy, injection of corticosteroid into the piriformis muscle may be tried, as well as infiltration with botulinum toxine A. Laparoscopic exploration with decompression of the sacral plexus or eventually partial resection of the piriformis muscle may be undertaken as a last resort.

## Pudendal Nerve Entrapment - Treatment

There are many treatment options depending on the cause of the pudendal neuropathy. One must keep in mind, that reduction or stopping association of medical pain treatment may be the first step in recovery of urinary functions, since most pain killers present side effects on bladder and sphincters control.

Because excessive tension (spasm) in the striated muscles of the pelvic floor appears to be common to most of the pelvic pain syndromes, treatment options always include pelvic floor physical therapy to relax pelvic floor muscles and pain medications. Trial of pudendal nerve blocks with botulinum toxine A is a real option [9], but bilateral infiltration must be done carefully to avoid urinary and fecal incontinence.

Where medical treatments are not successful in relieving symptoms, surgical treatments may be tried. In refractory pain situation, surgical treatment is a well know option. Robert has described the transgluteal approach for neurolysis of the PN at the infrapiriform canal and transection of the sacrospinal ligament [10]. Shafik on the other hand attempted to decompress the PN by following a perineal para-anal pathway. His dissection follows the inferior rectal nerve to the Alcock’s canal [11] and focused on the Alcock’s canal itself. The laparoscopic transperitoneal approach to the PN focuses mainly on the proximal portion of the PN but at the same time of the sacral nerves roots [12].

However surgical decompression of the nerve may not be effective in case of irreversible damage to the nerve, as may be seen in longstanding poorly controlled diabetes mellitus, or surgical damage or even destruction of the PN. Neurectomy is a possible option, but induces systematical numbness in corresponding nerve(s) distribution and exposes the patient to risk for incontinence if performed bilaterally and to risk for neuromas while duplicating the pre-existing pain level. Taking into consideration the underlying cause, location, and type of pain, attempts at conservative procedure should almost always be considered before moving to destructive procedures. It is well accepted that the actual state of the art neurosurgical treatment of peripheral neuropathic pain is neuromodulation that is an important part of the continuum of managing chronic intractable pain. It is hard to imagine contemporary pain management without this modality of treatment. Neuromodulation procedures are particularly attractive because they are nondestructive and reversible. The idea of achieving pain relief by electrically stimulating the dorsal columns of the spinal cord (spinal cord stimulation - SCS) was derived from the gate control theory by Melzack and Wall in 1965 [13].

Because sacral nerve stimulation does not permit neuromodulation of all pudendal afferent fibers together, it has not been considered to be a real therapeutic option for pudendal neuralgia in the international medical literature [14, 15]. To permit stimulation of the PN itself, the Bion® microstimulator was developed and designed to be implanted by perineal access adjacent to the pudendal nerve using a custom blunt dissector and cannula [16]. The main disadvantage of this device is the high risk for migration since implantation does not secure the device to any fixed anatomical structures. Spinelli has also reported about the technique of implantation of a tined lead near the PN by perineal or posterior approach [17], but with the same high risk for lead migration, dislocation or even cable breakage. The laparoscopic technique of implantation called “LION procedure” [18], enable a safe and reproducible implantation of a multiple channel electrode in direct contact to the PN within the protection of the pelvis. Fixation of the lead to neighbor endopelvic structures may prevents electrode dislocation, migration or cable breakage, but required general anesthesia and surgery. Neuromodulation of the SNR #2-4/5 has major advantage to permit simultaneously a pain control as a bladder control, especially for bladder overactivity.

## Conclusion

In PN entrapment, the main symptom is pain and the main objective of the treatment is control of pain. Nevertheless, physicians have to look after bladder dysfunctions especially bladder hypotonia and bladder overactivity not least because of risk for secondary kidneys damages and bladder overdilatation. Therefore clinical examination must combine gynecological, urological and neurological aspects – all knowledge contained in neuropelveology - but also urodynamic testing. It must be remember that, even when the awareness about pudendal neuralgie is still underestimated, the diagnosis of pudendal neuralgia is often overdiagnosed. Not only pathologies of the PN may induce pudendal pain, but all pathologies of the pudendal fibers from the brain to the peripheral PN distribution may induce such problems. Associated symptoms and pelvic dysfunctions may orient the diagnosis: PN entrapment must be suspected only in isolated pudendal pain while irradiation in sacral dermatomes (bottom, lower back, lower extremities) and bladder dysfunctions lead more to the diagnosis of a radiculopathies or a pathology of the central nervous system.

## References

[1] Reitz A, Schmid DM, Curt A, Knapp PA, Schurch B (2003). Afferent fibers of the pudendal nerve modulate sympathetic neurons controlling the bladder neck. Neurourol Urodyn..

[2] Roberts RO, Lieber MM, Bostwick DG, Jacobsen SJ (1997). A review of clinical and pathological prostatitis syndromes. Urology.

[3] Virseda Chamorro M, Salinas-Casdo J, Zarza-Lucianez D, Mendez-Rubio S, Pelaquim H, Esteban-Fuertes M (2012). Participation of the pudendal innervation in the detrusor overactivity of the detrusor and in the overactive bladder syndrome. Actas Urol Esp.

[4] Verdeja AM, Elkins TE, Odoi A, Gasser R, Lamoutte C (1995). Transvagnal sacropsinous colpopexy: anatomic landmarks to be aware of to minimize complications. Am J Obstet Gynecol.

[5] Debodinance P, Amblard J, Fatton B, Cosson M, Jacquetin B (2007). The prosthetic kits in the prolapsed surgery: is it a gadget?. J Gynecol Obstet Biol Reprod.

[6] Possover M, Lemos N (2011). Risks, symptoms, and management of pelvic nerve damage secondary to surgery for pelvic organ prolapse: a report of 95 cases. Int Urogynecol J..

[7] Moosey JJ (1987). Conus medullaris nerve root avulsions. J Neurosurg.

[8] Possover M (2010). New surgical evolutions in management of sacral radiculopathies. Surg Technol Int.

[9] Peng PW, Tumber PS (2008). Ultrasound-guided interventional procedures for patients with chronic pelvic pain - a description of techniques and review of literature. Pain Physician.

[10] Robert MI, Brunet C, Faure A, Lehur PA, Labat JJ, Bensigignor M (1993). La chirurgie du nerf pudendal lors de certaines algies perineales: evolution et resultats. Chirurgie.

[11] Shafik A (1992). Pudendal canal decompression in the treatment of idiopatic fecal incontinence. Dig Surg.

[12] Possover M (2009). Laparoscopic management of endopelvic etiologies of pudendal pain in 134 consecutive patients. J Urol.

[13] Melzack R, Wall P (1965). Pain mechanisms: a new theory. Science.

[14] Alo KM, Holsheimer J (2002). New trends in neuromodulation for the management of neuropathic pain. Neurosurgery.

[15] Peeren F, Hoebeke P, Everart K (2005). Sacral nerve stimulation: interstim therapy. Expert rev Med Devices.

[16] Bosch JLHR (2005). The Bion device: a minimally invasive implantate ministimulator for pudendal nerve neuromodulation in patients with detrusor overactivity incontinence. Urol Clin N Am.

[17] Spinelli M, Malaguti S, Giardiello G, Lazzeri M, Tarantola J, Van Den Hombergh U (2005). A new minimally invasive procedure for pudendal nerve stimulation to treat neurogenic bladder: description of the method and preliminary data. Neurourol Urodyn.

[18] Possover M (2010). The laparoscopic implantation of neuroprothesis to the sacral plexus for therapy of neurogenic bladder dysfunctions after failure of percutaneous sacral nerve stimulation. Neuromodulation.

